# Baby Buddy App for Breastfeeding and Behavior Change: Retrospective Study of the App Using the Behavior Change Wheel

**DOI:** 10.2196/25668

**Published:** 2021-04-15

**Authors:** Loretta M Musgrave, Alison Baum, Nilushka Perera, Caroline SE Homer, Adrienne Gordon

**Affiliations:** 1 Centre for Midwifery, Child and Family Health Faculty of Health University of Technology Sydney Ultimo NSW Australia; 2 Charles Perkins Centre Faculty of Medicine and Health University of Sydney Camperdown NSW Australia; 3 Best Beginnings London United Kingdom; 4 Burnet Institute Melbourne VIC Australia; 5 Royal Prince Alfred Hospital Sydney Local Health District NSW Health Camperdown NSW Australia

**Keywords:** breastfeeding, app, digital health, smartphone app, behavior change wheel, digital behavior change intervention

## Abstract

**Background:**

Breastfeeding plays a major role in the health of mothers and babies and has the potential to positively shape an individual’s life both in the short and long term. In the United Kingdom (UK), although 81% of women initiate breastfeeding, only 1% of women breastfeed exclusively to 6 months as recommended by the World Health Organization. In the UK, women who are socially disadvantaged and younger are less likely to breastfeed at 6 to 8 weeks postpartum. One strategy that aims to improve these statistics is the Baby Buddy app, which has been designed and implemented by the UK charity Best Beginnings to be a universal intervention to help reduce health inequalities, including those in breastfeeding.

**Objective:**

This study aimed to retrospectively examine the development of Baby Buddy by applying the Behavior Change Wheel (BCW) framework to understand how it might increase breastfeeding self-efficacy, knowledge, and confidence.

**Methods:**

Retrospective application of the BCW was completed after the app was developed and embedded into maternity services. A three-stage process evaluation used triangulation methods and formalized tools to gain an understanding of the potential mechanisms and behaviors used in apps that are needed to improve breastfeeding rates in the UK. First, we generated a behavioral analysis by mapping breastfeeding barriers and enablers onto the Capability, Opportunity, and Motivation-Behavior (COM-B) system using documents provided by Best Beginnings. Second, we identified the intervention functions and policy categories used. Third, we linked these with the behavior change techniques identified in the app breastfeeding content using the Behavior Change Techniques Taxonomy (BCTTv1).

**Results:**

Baby Buddy is a well-designed platform that could be used to change breastfeeding behaviors. Findings from stage one showed that Best Beginnings had defined breastfeeding as a key behavior requiring support and demonstrated a thorough understanding of the context in which breastfeeding occurs, the barriers and enablers of breastfeeding, and the target actions needed to support breastfeeding. In stage two, Best Beginnings had used intervention and policy functions to address the barriers and enablers of breastfeeding. In stage three, Baby Buddy had been assessed for acceptability, practicability, effectiveness, affordability, safety, and equity. Several behavior change techniques that could assist women with decision making around breastfeeding (eg, information about health consequences and credible sources) and possibly affect attitudes and self-efficacy were identified. Of the 39 videos in the app, 19 (49%) addressed physical capabilities related to breastfeeding and demonstrated positive breastfeeding behaviors.

**Conclusions:**

Applying a theoretical framework retrospectively to a mobile app is possible and results in useful information to understand potential health benefits and to inform future development. Future research should assess which components and behavioral techniques in the app are most effective in changing behavior and supporting breastfeeding.

## Introduction

A healthy start to life is crucial for improving life-long health outcomes [1,2]. Despite universal public funding for pregnancy care and targeted antenatal and postnatal programs, the United Kingdom (UK) has large inequalities in perinatal outcomes for women and children from minority ethnic communities, those who are socially disadvantaged, or those who become pregnant in their teenage years [3]. Breastfeeding is well recognized to positively impact and shape the lives of both the mother and baby in the short and long term. Global scaling up of breastfeeding interventions is needed to improve the rates of breastfeeding in all countries, which includes the provision of support to all women [1,2].

Breastmilk is nutritionally balanced and helps protect infants and children from infections [1]. There are risks associated with not breastfeeding in high-income, middle-income, and low-income countries [1]. A meta-analysis of six high-quality studies showed that “ever breastfeeding” (infants who have breastfed at least once) was associated with a 36% reduction in sudden infant death (95% CI 19%-49%) [2]. Breastfed babies have a lower chance of childhood leukemia and allergies, and are less likely to develop diabetes or become overweight when they are older [4]. Breastfeeding also benefits mothers, and it is associated with a lower risk of developing breast and ovarian cancer, osteoporosis, diabetes, and cardiovascular disease [2]. A longer period of breastfeeding is also associated with a reduction in the mother's odds of overweight or obesity (95% CI 22-30) [4].

The UK National Infant Feeding Survey (2010) showed that although 81% of women initiated breastfeeding, 34% of babies received any breastmilk at 6 months (only 1% were exclusively breastfed), and the country ranks lowest in the world for breastfeeding at 12 months of age [2,3]. The most recent aggregate breastfeeding rate for England (Quarter 3 of 2019/20) at 6 to 8 weeks was 48.2% (CI 47.9%-48.5%) [5]. As a response to low breastfeeding rates, the UK Public Health England in collaboration with UNICEF UK, has produced several policies and resources in line with the “baby friendly initiative.” It is hoped that initiatives that promote breastfeeding will augment women’s and children’s health and support maternal-infant bonding [6,7].

In 2007, Best Beginnings charity in the UK co-designed digital video discs (DVDs) to support breastfeeding initiation, motivation, and duration, with a focus on benefits and acknowledgement of challenges. The resources were developed with parents, the UK Department of Health, and UNICEF UK. Since the 2008 launch, over 2 million copies of the DVD have been distributed. In 2014, with changing technology, the charity embedded this breastfeeding content into Baby Buddy, a smartphone app. Pregnant women are now more likely to find pregnancy apps useful sources of information and support compared with DVDs or written material [8-12]. This trend toward the use of smartphones provides an opportunity to reach those women who are less likely to engage with health care providers or are yet to do so [13,14].

Baby Buddy was designed to focus on the window of opportunity from preconception to 6 months of age, in which the foundations for a healthy childhood are laid [15]. The app is free, available on the National Health Service Library, embedded into maternity and early care pathways, and endorsed by organizations, including the Royal College of Midwives and the Royal College of Obstetricians and Gynaecologists, and it can be easily accessed on both Android and iOS devices. Baby Buddy is intended to be used by parents of all backgrounds and to be particularly engaging for those who may have difficulty connecting with health services owing to language, age, culture, or socioeconomic barriers. Baby Buddy has been designed to appeal to younger women and includes a user-designed interactive avatar as a “gaming” element. The app aims to build confidence and self-efficacy and promote good parental-infant bonding and attachment. It contains over 300 videos, including all videos from the “From bump to breastfeeding” DVD, and provides engaging and interactive daily information to support healthy behaviors including breastfeeding. The app intends to enhance the link between parents and health care providers and promotes better engagement, communication, and shared decision making with parents [16].

The most recent published evaluation of Baby Buddy, the BaBBLeS study (Bumps and Babies Longitudinal Study), measured maternal self-efficacy as the primary outcome. The authors found that there were no differences in maternal self-efficacy outcomes. However, they did perform a post-hoc analysis of breastfeeding and documented a significant increase in “any breastfeeding” at 1 month (odds ratio [OR] 3.08, 95% CI 1.49-6.35) and in “exclusive breastfeeding” at 3 months (OR 1.79, 95% CI 1.02-3.16) [16]. Further data from Norfolk did demonstrate an increase in maternal self-efficacy for parents using the Baby Buddy app [17].

With this data demonstrating potential behavior change and increased breastfeeding with the use of the Baby Buddy app, further understanding was sought regarding which components of the design and development of the app might have contributed to these results. The Behavior Change Wheel (BCW) and the associated Behavior Change Techniques Taxonomy (BCTTv1) provide a systematic approach that acknowledges the importance of behavioral theory in the design and evaluation of interventions. The BCW has three interrelated concentric layers. The inner layer (Capability, Opportunity, and Motivation-Behavior [COM-B] model) helps understand the behavior that needs to be changed. The middle layer consists of the following possible interventions that could be used to facilitate behavior change: restrictions, education, persuasion, incentivization, coercion, training, enablement, modeling, and environmental restructuring. The outer layer of the wheel assists in identifying which policy opportunities could be utilized to support the delivery of the chosen interventions [18]. Finally, the BCTTv1 is a complementary tool that helps further identify which behavior change techniques could help deliver the intervention functions identified [18]. The BCW has previously been retrospectively applied to other mobile health interventions successfully [19-21]. This study aimed to retrospectively examine the development of Baby Buddy and apply the BCW framework to understand how it might increase breastfeeding self-efficacy, knowledge, and confidence.

## Methods

### Overview

**Figure 1:**
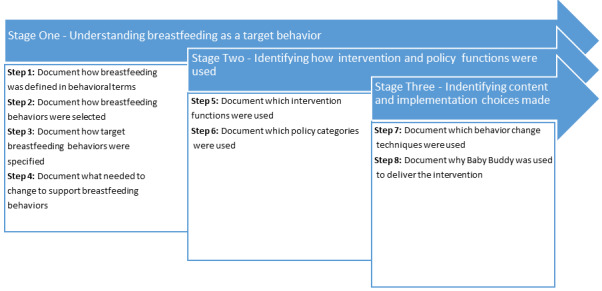
Process of applying the Behavior Change Wheel to the Baby Buddy app.

We evaluated the development of Baby Buddy with the BCW and its associated taxonomy using a prespecified three-stage process (Figure 1). The research was conducted between November 2017 and December 2018. The research team was given access to all reports, market research, and interview and focus group findings prepared by Best Beginnings to inform the design of Baby Buddy (Multimedia Appendix 1). Guide books containing worksheets were used to deconstruct and retrospectively analyze the development process and the breastfeeding components within the Baby Buddy app [18,22]. Data extraction was performed by one reviewer (LMM) and then checked by a second (AG). They met fortnightly to share and discuss the findings. This was achieved by cross-checking coding, interpretation, and mapping. Any discrepancy was resolved by discussion, and further analysis or content review was undertaken if necessary.

### Stage One: Understanding Breastfeeding as a Target Behavior

This stage aimed to assess the in-depth understanding of breastfeeding as a target behavior in the development of the app and the context in which it occurs. Barriers and enablers to target behaviors were identified in the provided data (survey, interview, and focus group reports), and then, these were mapped to the COM-B tool [18].

### Stage Two: Identifying How Intervention and Policy Functions Were Used

This stage determined the aspects included in Baby Buddy and if they could influence breastfeeding behavior. The middle layer of the BCW was used by the research team to map which “intervention” components could address the barriers and enablers to breastfeeding (restrictions, education, persuasion, incentivization, coercion, training, enablement, modeling, and environmental restructuring) [18]. We then coded these findings using the Theoretical Domains Framework (TDF) (knowledge, cognitive and interpersonal skills, memory, attention and decision-making processes, optimism, beliefs about consequences, intentions, goals, emotions, and social influences) [18]. The outer layer of the BCW was used to map policy categories (eg, policies, guidelines, fiscal measures, service provision, legislation, regulation, communication, and environmental opportunities) [18].

### Stage Three: Identifying Content and Implementation Choices Made

This stage identified evidence of the use of behavioral change techniques (BCTs) within the design of Baby Buddy. We used the APEASE criteria as defined in the BCW (affordability, practicability, effectiveness, affordability, safety, and equity) [18,22]. These steps provided insights into how the content was developed and implemented and to understand the choices made by Best Beginnings as the project progressed. We also described the “active” ingredients that were used in the breastfeeding intervention using the BCTTv1 tool [18,22]. To do this, we viewed and reviewed 39 videos, eight glossary words (“What does that mean?”), and 20 Baby Buddy–generated responses to breastfeeding questions (“Ask me”). We marked the BCTTv1 tool for each technique found in each piece of information reviewed (videos, glossary words, and generated responses).

## Results

### Stage One: Understanding Breastfeeding as a Target Behavior

#### Step 1: How Breastfeeding was Defined in Behavioral Terms

Best Beginnings defined breastfeeding as a key behavior requiring more support and demonstrated a thorough understanding of the context in which breastfeeding occurs based on the following sources of evidence: (1) The Infant Feeding Survey (2010) [3]; (2) World Health Organization Global Strategy for Infant and Young Child Feeding (Breastfeeding Manifesto) [23]; (3) Tackling health inequalities in infant and maternal health outcomes [15]; (4) Focus On: A Proportionate Approach to Priority Populations [24]; (5) Fair Society, Healthy Lives – Strategic Review of Health Inequalities in England post-2010 [25]; and (6) The Foundation Years: preventing poor children becoming poor adults [26].

#### Step 2: How Breastfeeding Behaviors Were Selected

Best Beginnings selected specified target actions that were needed to support breastfeeding. They undertook extensive consultation with stakeholders, including the UK Department of Health, UNICEF, and women and their families. A multidisciplinary team approach was adopted in the creation of the steering committee. The following six target behaviors to support breastfeeding were identified as a priority by Best Beginnings: (1) Advising on commencing breastfeeding; (2) Giving information on correct positioning and attachment for breastfeeding; (3) Knowing how to express breast milk; (4) Knowing what is normal in the first few months of breastfeeding; (5) Knowing how to overcome breastfeeding challenges; and (6) Planning to breastfeed for 6 months or more.

#### Step 3: How Target Breastfeeding Behaviors Were Specified

Breastfeeding behaviors were described with who, what, when, where, how often, and with whom (Table 1). Best Beginnings utilized mixed method techniques to better understand the barriers and enablers affecting inequity, disparity, and intergenerational disadvantage (Multimedia Appendix 1). Health care professionals, parents, and families were engaged as co-creators at all stages and were instrumental in app development, implementation, evaluation, and promotion [16,27-30].

**Table 1:** Specifying breastfeeding as a target behavior [22].

Question	Response
*Who* needs to perform the behavior?	Women, with a focus on young women under 25 years intending to breastfeed
*What* does the person need to do differently to achieve the desired change?	Offer breast first
*When* will they do it?	Within the first hour of birth and then for every feed demanded
*Where* will they do it?	At the birthplace and then anywhere they choose to feed the infant
*How often* will they do it?	Every feed
*With whom* will they do it?	With the support of staff initially and then independently with the support of family and friends or professionals if required

#### Step 4: Changes Needed to Support Breastfeeding Behaviors

We found evidence to support that the constructs of capability, opportunity, and motivation were explored as described below.

##### Physical and Psychological Capability

Best Beginnings explored social norms, peer influence, and the value of social support in sustaining breastfeeding. For example, women were asked to discuss breastfeeding in the context of their roles in their families, the presence or absence of support, the influences of cultural values, and the impact of migration, isolation, and loneliness. Peer and clinical support, demonstrations, practice, and feedback were seen as important to enable women to breastfeed. Perceived barriers, such as difficulties positioning and attaching, low milk production (physical capability), fear of failure, and anxiety/depression (psychological capability), were identified as needing to be addressed by the intervention functions (Multimedia Appendix 2).

##### Physical and Social Opportunity

Support was identified as the primary enabler for both physical and social opportunity to breastfeed. Clinical/specialist, peer, community, and technology supports (apps, social media, and online resources) were documented as facilitators for breastfeeding. Best Beginnings sought to understand environmental factors that may help, interfere, or prevent breastfeeding efforts. Economic barriers and the physical environment were discussed, and there were several themes related to challenges in finding a way to initiate and maintain breastfeeding behaviors in the context of roles as employees, mothers, and partners (Multimedia Appendix 2).

##### Reflective and Automatic Motivation

Reviewed data demonstrated that motivation is best facilitated by early planning, goal setting, and positive belief reinforcement. Peer support normalizes the challenges of breastfeeding and encourages self-determination. Best Beginnings documented support as crucial to helping alleviate negative thoughts or low confidence. Self-efficacy to change beliefs and habits, and low health literacy barriers were explored to assess the ability of individuals to act on health advice and planned care and to uncover culturally specific values that may improve interventions in specific target groups (Multimedia Appendix 2).

### Stage Two: Identifying How Intervention Functions Were Used

#### Step 5: Intervention Functions That Were Used

Intervention functions were able to be identified in the video content for Baby Buddy, which included the lead information and education resource within the app. The most common functions were education, training, and modeling. Mapping of the breastfeeding video content to the BCW (COM-B, TDF, and intervention functions) is shown in Multimedia Appendix 2. Further analysis of each video containing breastfeeding content (39 videos) is shown in Table 2. The complete analysis of all breastfeeding items, including eight glossary words (“What does that mean?”) and 20 Baby Buddy–generated responses to breastfeeding questions (‘Ask me’), using COM-B, is shown in Multimedia Appendix 3.

**Table 2:** Mapping of breastfeeding video content to the Capability, Opportunity, and Motivation-Behavior (COM-B) tool.

Video title	Capability			Opportunity			Motivation		
	Physical	Psychological	Social		Physical	Reflective		Automatic	
Breastfeeding as a young mum	Yes	Yes	Yes		Yes	Yes		Yes	
A practical choice	Yes	Yes	Yes		Yes	Yes		Yes	
Feelings about breastfeeding	Yes	Yes	Yes		Yes	Yes		Yes	
What’s so good about breastfeeding?	No	Yes	Yes		Yes	Yes		Yes	
What if I bottle fed before?	Yes	Yes	Yes		Yes	Yes		Yes	
Asking for help to get started	Yes	Yes	Yes		Yes	No		No	
What will my partner think?	No	No	Yes		No	Yes		No	
Your first milk - colostrum	Yes	Yes	No		No	Yes		Yes	
Your baby’s first feed	Yes	Yes	Yes		Yes	No		Yes	
Skin to skin	Yes	Yes	Yes		Yes	No		Yes	
Good positioning tips from a midwife	Yes	No	Yes		No	No		Yes	
Getting the position right	Yes	No	No		Yes	No		Yes	
Good positioning demonstration	Yes	Yes	No		Yes	Yes		No	
Keeping your baby close	Yes	Yes	Yes		Yes	Yes		Yes	
How dads can help? - Lenny	No	No	Yes		Yes	No		No	
Breastfeeding out and about	No	Yes	Yes		No	Yes		Yes	
When and how often should I feed my baby?	Yes	Yes	Yes		Yes	Yes		Yes	
How dads can help? - Andy	No	No	Yes		Yes	Yes		No	
Where can I find support?	Yes	Yes	Yes		Yes	Yes		No	
Overcoming mastitis	Yes	Yes	Yes		Yes	Yes		Yes	
Support from health professionals	Yes	Yes	Yes		Yes	Yes		Yes	
Some common challenges	Yes	Yes	No		Yes	Yes		No	
Good and bad attachment graphic	Yes	No	No		Yes	No		No	
Breastfeeding to a year and beyond	Yes	Yes	Yes		Yes	Yes		Yes	
Why breastfeed for at least six months?	Yes	Yes	Yes		Yes	Yes		Yes	
Breastfeeding and weening	Yes	Yes	Yes		Yes	Yes		Yes	
Why express?	Yes	Yes	Yes		Yes	Yes		Yes	
How to hand express?	Yes	Yes	No		Yes	Yes		Yes	
How to use a breast pump?	Yes	No	No		Yes	Yes		No	
Expressing when you’re back at work	Yes	Yes	No		Yes	Yes		No	
Storing and using expressed breast milk	No	No	No		Yes	Yes		No	
Early challenges with expressing milk	Yes	Yes	No		Yes	Yes		No	
Your breast milk	Yes	Yes	Yes		Yes	Yes		Yes	
How skin-to-skin contact can help you express?	Yes	Yes	Yes		Yes	Yes		Yes	
Using a breast pump	Yes	No	Yes		Yes	Yes		No	
Expressing with a breast pump and storing your milk	Yes	Yes	Yes		Yes	Yes		Yes	
Colostrum - your baby’s first food	Yes	Yes	No		Yes	No		Yes	
Signs your baby is ready to feed independently	No	Yes	Yes		Yes	Yes		No	
Breastfeeding twins or triplets	Yes	No	No		Yes	Yes		No	

#### Step 6: Policy Categories That Were Used

Findings support that the Baby Buddy app and its embedding process were designed to complement maternity and postnatal health service and policy [28]. It has been endorsed by the Department of Health, Faculty of Public Health, Royal Colleges of Paediatrics and Child Health, obstetricians and gynecologists, midwives, psychiatrists, speech and language therapists, community practitioners, Health Visitors Association, and Institute of Health Visiting. The content of Baby Buddy was co-created with parents and in consultation with policy stakeholders, for example, representatives from Royal Colleges and the Department of Health. No content is uploaded to Baby Buddy until representatives of all partners have given their approval.

### Stage Three: Identifying How Content and Implementation Choices Were Made

#### Step 7: How Behavior Change Techniques Were Used

Identification of BCTs was achieved by applying the BCTTv1 to the content of the app. After each piece of content was categorized using broad intervention categories, further analysis was carried out to identify exactly which BCTs were used (Multimedia Appendix 2). These were then documented and specific details were given. For example, of the 39 videos in the app, 19 (49%) addressed physical capabilities related to breastfeeding and demonstrated positive breastfeeding behaviors.

#### Step 8: Rationale For Using the Baby Buddy App as the Mode of Delivery

The APEASE criteria were used to evaluate if Best Beginnings had undertaken activities to ascertain acceptability, practicability, effectiveness, affordability, safety, and equity when moving breastfeeding content to a mobile app. The evidence was analyzed and judged against the previous DVD-based breastfeeding intervention, “Bump to breastfeeding.” Baby Buddy met the APEASE criteria for a viable digital intervention suitable for further testing, development, and implementation (Multimedia Appendix 4). In addition, it was noted that in transitioning from DVD to a mobile app, Best Beginnings used the Kotter eight-step process to guide implementation. Kotter methodology, developed for change management, involves the following eight steps: (1) creating a sense of urgency, (2) building a guiding coalition, (3) forming strategic vision and initiatives, (4) enlisting a volunteer army, (5) enabling action by removing barriers, (6) generating short-term wins, (7) sustaining acceleration, and (8) instituting change [31,32].

## Discussion

### Principal Findings

Baby Buddy maps retrospectively well to the BCW. This may explain why there have been positive results in recent studies [28,29]. Key factors that set the development of this particular pregnancy app apart from many others are the genuine co-design and the use of BCTs most obviously through the included video content.

The use of participatory engagement and co-creation methods in the development of Baby Buddy are two design techniques that have positively influenced decision making, attitudes, and self-efficacy concerning breastfeeding, particularly among those who are socially disadvantaged and younger. We identified several BCTs used in Baby Buddy that could assist women with decision making around breastfeeding (eg, BCT 5.1 Information about health consequences and 9.1 Credible source). BCTs that influence attitudes and self-efficacy were also identified (eg, BCT 5.3 Information about social and environmental consequences and 13.2 Framing/reframing).

### Strengths and Limitations

This study has several strengths. First, it was performed independent of the development team, using a best practice behavior change framework (BCW) as a guide. Second, content mapping to the BCW was conducted by two independent content experts (a midwife and a neonatologist). These two research team members located in Australia were not employed by Best Beginnings and did not have any financial incentive. Third, retrospective alignment of the BCW tools and BCTs enabled the research team to identify potential opportunities to use BCTs for the future development of Baby Buddy to increase effectiveness. Fourth, our study supports the work of Thomson and Crossland who conducted a mixed methods evaluation using the BCW to identify components that support infant feeding in North West UK [29]. They identified peer support as a facilitator for increasing mothers’ knowledge and building confidence [29]. Finally, we also identified the use of peer-to-peer content as beneficial for breastfeeding as it normalizes breastfeeding and encourages self-determination. Baby Buddy has both of these attributes in the content. Like the work of Crossland et al, our study concluded that Baby Buddy is a supportive parenting resource that could be scaled for impact [28].

A key limitation of this work is the retrospective application of the BCW. Retrospective mapping of the BCW to the app development process was complex and subjective, and relied on Best Beginnings providing multiple development documents. There was a large volume of qualitative reports supplied to us from Best Beginnings that had been collected from many sources and not presented with later academic review in mind.

Using the BCW has inherent coding, interpretation, and application limitations. However, like other studies, we do believe that there is benefit in “retrofitting” interventions to the BCW even though it may have not been used in the design phase [33-35]. Prospective analysis of the app development using the BCW and scientific research would potentially result in a higher quality behavior change intervention tool; however, Baby Buddy was not primarily designed to change behavior and was rather designed as a resource to inform and empower pregnant women.

A secondary limitation is that the evaluation tools we used were designed for text rather than video content. From our assessment, videos within an app appear to be a powerful influence to support behavior change in breastfeeding. The videos take a “show how” approach rather than a didactic “tell to” approach and feature a mixture of experts, support parents, and peer-to-peer voices. However, as the BCW tools were not designed for video discourse analysis specifically, they may miss some of the nuances in video content (eg, gesturing, body language, and tone). Our findings have identified potential areas for improvement in future iterations of the app, and this is useful information given that the app is constantly being improved.

### Conclusion

Our work highlights that applying a theoretical framework retrospectively to a mobile health app is possible and results in useful information to understand potential health benefits and to inform future development. To assess the true impact of behavior change frameworks in the design of mobile health apps, high-quality research that measures formative, process, and clinical outcomes for health behaviors is needed. Further development of Baby Buddy as a universal intervention to reduce health inequalities requires robust prospective research that considers effects on the rate and duration of exclusive breastfeeding.

## Appendix

Multimedia Appendix 1 Reports supplied by Best Beginnings.

Multimedia Appendix 2 Using the Behavior Change Wheel (BCW) to analyze breastfeeding video content in the Baby Buddy app.

Multimedia Appendix 3 Complete analysis of all breastfeeding items.

Multimedia Appendix 4 APEASE (affordability, practicability, effectiveness, affordability, safety, and equity) criteria.

## Abbreviations

APEASE: affordability, practicability, effectiveness, affordability, safety, and equity
BCT: behavior change technique
BCTTv1: Behavior Change Techniques Taxonomy
BCW: Behavior Change Wheel
COM-B: Capability, Opportunity, and Motivation-Behavior
DVD: digital video disc
OR: odds ratio
TDF: Theoretical Domains Framework
UK: United Kingdom

## References

[1] Rollins NC, Bhandari N, Hajeebhoy N, Horton S, Lutter CK, Martines JC, Piwoz EG, Richter LM, Victora CG (2016). Why invest, and what it will take to improve breastfeeding practices?. The Lancet.

[2] Victora CG, Bahl R, Barros AJD, França GVA, Horton S, Krasevec J, Murch S, Sankar MJ, Walker N, Rollins NC (2016). Breastfeeding in the 21st century: epidemiology, mechanisms, and lifelong effect. The Lancet.

[3] McAndrew FT, Fellows L, Large A, Speed M, Renfrew M (2012). Infant Feeding Survey 2010. UK Data Service.

[4] Horta BL, Loret de Mola C, Victora CG (2015). Long-term consequences of breastfeeding on cholesterol, obesity, systolic blood pressure and type 2 diabetes: a systematic review and meta-analysis. Acta Paediatr.

[5] (2020). Breastfeeding prevalence at 6-8 weeks after birth (Experimental Statistics). Public Health England.

[6] Breastfeeding in England. Unicef United Kingdom.

[7] Protecting Health and Saving Lives: A Call to Action. Unicef United Kingdom.

[8] Kennedy R, Mullaney L, Reynolds C, Cawley S, McCartney D, Turner M (2017). Preferences of women for web-based nutritional information in pregnancy. Public Health.

[9] Tang K, Gerling K, Chen W, Geurts L (2019). Information and Communication Systems to Tackle Barriers to Breastfeeding: Systematic Search and Review. J Med Internet Res.

[10] Wang N, Deng Z, Wen LM, Ding Y, He G (2019). Understanding the Use of Smartphone Apps for Health Information Among Pregnant Chinese Women: Mixed Methods Study. JMIR Mhealth Uhealth.

[11] O'Higgins A, Murphy OC, Egan A, Mullaney L, Sheehan S, Turner MJ (2014). The use of digital media by women using the maternity services in a developed country. Ir Med J.

[12] Rodger D, Skuse A, Wilmore M, Humphreys S, Dalton J, Flabouris M, Clifton VL (2013). Pregnant women’s use of information and communications technologies to access pregnancy-related health information in South Australia. Aust. J. Prim. Health.

[13] Hearn L, Miller M, Lester L (2014). Reaching perinatal women online: the Healthy You, Healthy Baby website and app. J Obes.

[14] Olander EK, Darwin ZJ, Atkinson L, Smith DM, Gardner B (2016). Beyond the 'teachable moment' - A conceptual analysis of women's perinatal behaviour change. Women Birth.

[15] (2010). Tackling health inequalities in infant and maternal health outcomes. Department of Health.

[16] Deave T, Ginja S, Goodenough T, Bailey E, Piwek L, Coad J, Day C, Nightingale S, Kendall S, Lingam R (2019). The Bumps and BaBies Longitudinal Study (BaBBLeS): a multi-site cohort study of first-time mothers to evaluate the effectiveness of the Baby Buddy app. Mhealth.

[17] (2020). Self Care Project for Parents using Just One Norfolk website and the Baby Buddy app (Final report). Best Beginnings.

[18] Michie S, Atkins L, West R (2014). The Behaviour Change Wheel: A Guide To Designing Interventions.

[19] Brown HM, Bucher T, Collins CE, Rollo ME (2019). A review of pregnancy iPhone apps assessing their quality, inclusion of behaviour change techniques, and nutrition information. Matern Child Nutr.

[20] Gould GS, Bar-Zeev Y, Bovill M, Atkins L, Gruppetta M, Clarke MJ, Bonevski B (2017). Designing an implementation intervention with the Behaviour Change Wheel for health provider smoking cessation care for Australian Indigenous pregnant women. Implement Sci.

[21] Tombor I, Shahab L, Brown J, Crane D, Michie S, West R (2016). Development of SmokeFree Baby: a smoking cessation smartphone app for pregnant smokers. Transl Behav Med.

[22] West R, Michie S (2016). A Guide to Development and Evaluation of Digital Behaviour Change Interventions in Healthcare.

[23] (2003). Global Strategy for Infant and Young Child Feeding. World Health Organization.

[24] (2015). Focus On: A Proportionate Approach to Priority Populations. Ontario Agency for Health Protection and Promotion.

[25] Marmot M, Bell R (2012). Fair society, healthy lives. Public Health.

[26] Field F (2010). The foundation years: preventing poor children becoming poor adults, the report of the Independent Review on Poverty and Life Chances. Digital Education Resource Archive.

[27] Cooper S (2015). App Pilot Evaluation Report: National in-app data & in-app data from Guys and St Thomas’ and Blackpool. Reporting period: 19 November 2014 to 19 May 2015.

[28] Crossland N, Thomson G, Moran VH (2019). Embedding supportive parenting resources into maternity and early years care pathways: a mixed methods evaluation. BMC Pregnancy Childbirth.

[29] Thomson G, Crossland N (2019). Using the behaviour change wheel to explore infant feeding peer support provision; insights from a North West UK evaluation. Int Breastfeed J.

[30] Powell S, Ali Z, Christie S, Apps J, Goouch K (2016). Report on the Evaluation of Baby Buddy M-Health Intervention with a focus on the GSTT Pilot Embedding Site. In.

[31] Kotter JLC (1996). Leading Change.

[32] The 8-Step Process For Leading Change. Kotter.

[33] Steinmo S, Fuller C, Stone SP, Michie S (2015). Characterising an implementation intervention in terms of behaviour change techniques and theory: the 'Sepsis Six' clinical care bundle. Implement Sci.

[34] Watkins K, Seubert L, Schneider CR, Clifford R (2016). Post hoc evaluation of a common-sense intervention for asthma management in community pharmacy. BMJ Open.

[35] Chiang N, Guo M, Amico KR, Atkins L, Lester RT (2018). Interactive Two-Way mHealth Interventions for Improving Medication Adherence: An Evaluation Using The Behaviour Change Wheel Framework. JMIR Mhealth Uhealth.

