# Supplementary material for: Baby Buddy App for Breastfeeding and Behavior Change: Retrospective Study of the App Using the Behavior Change Wheel
Source: JMIR Mhealth Uhealth. 2021 Apr 15;9(4):e25668. doi: 10.2196/25668 (PMC8085747; doi:10.2196/25668)
Supplement: Multimedia Appendix 1 [file mhealth_v9i4e25668_app1.docx]

**Multimedia Appendix 1. Reports supplied by Best Beginnings.**

1) Focus groups and interviews conducted by Alex Walley Research (2012) to test assumptions and concepts (unpublished observations).

2) Focus groups and interviews conducted by Alex Walley Research (2014) to test app proto-type (unpublished observations).

3) Focus groups conducted by Alex Walley Research (2014) post-launch

Qualitative research (unpublished observations).

4) App Pilot Evaluation Report: National in-app data & in-app data from Guys and St Thomas’ and Blackpool (2014-2015) [[1](#_ENREF_1)].

5) Report on the Evaluation of Baby Buddy M-Health Intervention with a focus on the GSTT Pilot Embedding Site (2016) [[2](#_ENREF_2)].

6) The BaBBLeS study (Bumps and BaBies Longitudinal Study) (2016) [[3](#_ENREF_3), [4](#_ENREF_4)]. Ethics approval from the West Midlands-South Birmingham Research Ethics Committee (NRES) (16/WM/0029) and the University of the West of England, Bristol, Research Ethics Committee (HAS.16.08.001).

7) Process evaluation using ethnographic methods (observations and interviews), surveys, monitoring, and outcome data analysis by the University of Central Lanchashire (2017) (unpublished observations). Ethics approval from The East Midlands-Nottingham 2 NRES Committee and the Science, Technology, Engineering, Medicine, and Health (STEMH) ethics sub-committee at the University of Central Lancashire (project no. 358) provided ethics approval for the study. Governance approval was granted by all relevant NHS trusts at each of the three sites.

8) Embedding Supportive Parenting Resources into Maternity and Early Years Care Pathways: a Mixed Methods Evaluation. Crossland N, Thomson G, Moran VH (2019) [[5](#_ENREF_5)]. Ethical approval was given by The East Midlands-Nottingham 2 NRES Committee (26th May 2015), and by the Science, Technology, Engineering, Medicine and Health (STEMH) ethics sub-committee at the University of Central Lancashire (project no. 358; 15th June 2015). Additionally, governance approval was granted by all relevant NHS organisations at each of the three study sites.

9) Self Care Project for Parents using Just One Norfolk website and the Baby Buddy app (Final report) (2020). The University of Essex Health and Care Research Service (HCRS) [[6](#_ENREF_6)].

**References**

1. Cooper S: **App Pilot Evaluation Report: National in-app data & in-app data from Guys and St Thomas’ and Blackpool (reporting period: 19 November 2014 to 19 May 2015)**. In*.*

2. Powell S, Ali Z, Christie S, Apps J, Goouch K: **Report on the Evaluation of Baby Buddy M-Health Intervention with a focus on the GSTT Pilot Embedding Site**. In*.* UK: Research Centre for Children, Families and Communities, Canterbury Christ Church University; 2016.

3. Deave T, Kendal S, Lingam R, Day C, Goodenough T, Bailey E, Ginja S, Nightingale S, Coad J: **A study to evaluate the effectiveness of Best Beginnings’ Baby Buddy phone app in England: a protocol paper**. *Primary Health Care Research &#x0026; Development* 2018:1-6.

4. Deave T, Ginja S, Goodenough T, Bailey E, Piwek L, Coad J, Day C, Nightingale S, Kendall S, Lingam R: **The Bumps and BaBies Longitudinal Study (BaBBLeS): a multi-site cohort study of first-time mothers to evaluate the effectiveness of the Baby Buddy app**. *mHealth* 2019, **5**:42.

5. Crossland N, Thomson G, Moran VH: **Embedding supportive parenting resources into maternity and early years care pathways: a mixed methods evaluation**. *BMC Pregnancy and Childbirth* 2019, **19**(1).

6. Service TUoEHaCR: **Self Care Project for Parents using Just One Norfolk website and the Baby Buddy app (Final report)**. In*.* UK: The University of Essex; 2020.
