# Supplementary material for: Baby Buddy App for Breastfeeding and Behavior Change: Retrospective Study of the App Using the Behavior Change Wheel
Source: JMIR Mhealth Uhealth. 2021 Apr 15;9(4):e25668. doi: 10.2196/25668 (PMC8085747; doi:10.2196/25668)
Supplement: Multimedia Appendix 2 [file mhealth_v9i4e25668_app2.docx]

**Multimedia Appendix 2. Using the Behavior Change Wheel (BCW) to analyze breastfeeding video content in the Baby Buddy app.**

| **COM-B** | **COM-B Component** | **Relevant Theoretical Domains Framework (TDF)** | **Description of what needs addressing in the intervention based on the data collected** | **Intervention functions** | **Behavior change techniques (BCTs) identified** | **Number of videos containing the BCTs** |
| --- | --- | --- | --- | --- | --- | --- |
| **Capability** | **Physical capability** | Physical skills | 1. Having the physiological ability to lactate | Education | 4.1 Instruction on how to perform the behavior | 19 |
|  |  |  | 2. Having the physical capability and skills to breastfeed and express milk | Training | 6.1 Demonstration of the behavior | 19 |
|  |  |  |  | Modelling | 7.1 Prompts/cues | 16 |
|  |  |  |  |  | 8.1 Behavioral practice/rehearsal | 18 |
|  |  |  |  |  | 9.1 Credible source | 19 |
|  | **Psychological capacity** | Knowledge | 1. Knowledge about the benefits of breastfeeding | Education | 1.2 Problem solving | 13 |
|  |  | Cognitive and interpersonal skills | 2. Knowledge of how to attach and position the baby for breastfeeding | Training | 2.3 Self-monitoring of behavior | 14 |
|  |  | Memory, attention and decision processes | 3. Ability to problem solve and make decisions related to breastfeeding | Modelling | 4.1 Instruction on how to perform the behavior | 17 |
|  |  | Behavioral regulation | 4. Belief in ability to produce required amount of breast milk and to breastfeed successfully | Persuasion | 5.1 Information about health consequences | 18 |
|  |  |  | 5. Increase confidence in ability to breastfeed | Enablement | 5.3 Information about social and environmental consequences | 13 |
|  |  |  | 6. Beliefs about the consequences of not breastfeeding e.g. regret, feeling a failure as a mother |  | 6.1 Demonstration of the behavior | 16 |
|  |  |  | 7. Conscious decision to breastfeed successfully |  | 7.1 Prompts/cues | 14 |
|  |  |  | 8. Increase ability to deal with emotions related to breastfeeding e.g. anxiety about capacity |  | 8.1 Behavioral practice/rehearsal | 16 |
|  |  |  |  |  | 9.1 Credible source | 17 |
| **Opportunity** | **Physical opportunity** | Environmental context and resources | 1. Perception that breastfeeding is difficult due to the unpredictable nature of demand feeding | Education | 4.1 Instruction on how to perform the behavior | 17 |
|  |  |  | 2. Having unlimited access to the well infant or demand feeding | Enablement | 7.1 Prompts/cues | 12 |
|  |  |  |  | Environmental restructuring | 12.1 Restructuring the physical environment | 9 |
|  |  |  |  |  | 12.2 Restructuring the social environment | 5 |
|  |  |  |  |  | 12.3 Avoidance/reducing exposure to cues for the behavior | 4 |
|  | **Social opportunity** | Social influences | 1. Perception that breastfeeding is difficult | Education | 2.3 Self-monitoring of behavior | 14 |
|  |  |  | 2. Encouragement from health care professionals | Persuasion | 5.1 Information about health consequences | 11 |
|  |  |  | 3. Support of friends, family and community | Modelling | 5.3 Information about social and environmental consequences | 14 |
|  |  |  | 4. Holds the belief that exclusive breastfeeding is socially acceptable and culturally normal | Environmental restructuring | 6.3 Information about other's approval | 9 |
|  |  |  |  |  | 7.1 Prompts/cues | 2 |
|  |  |  |  |  | 9.1 Credible source | 7 |
|  |  |  |  |  | 12.2 Restructuring the social environment | 6 |
| **Motivation** | **Reflective motivation** | Professional/  social role and identity | 1.Don't like the idea of needing or seeking help/belief that women should be able to breastfeed 'naturally' | Education | 5.5 Anticipated regret | 6 |
|  |  | Beliefs and capabilities | 2.Needing help with breastfeeding means there is a problem | Persuasion | 5.6 Information about emotional consequences | 16 |
|  |  | Optimism | 3.Disappointment with self if unable to breastfeed | Modelling | 11.2 Reduce negative emotions | 9 |
|  |  | Beliefs about consequences | 4.Believing that breastfeeding is the best method of feeding |  | 13.2 Framing/re-framing | 14 |
|  |  | Intentions | 5. Want to breastfeed this time as bottle fed last time |  | 13.5 Identity associated with changed behavior | 13 |
|  |  | Goals |  |  | 15.3 Focus on past success | 2 |
|  | **Automatic motivation** | Reinforcement | 1.Emotionally driven to breastfeed because of the benefits associated such as bonding and attachment | Education | 4.2 Information about antecedents | 10 |
|  |  | Emotion | 2. Fear of failing to breastfeed successfully | Incentivization | 5.1 Information about health consequences | 15 |
|  |  |  |  | Modelling | 5.2 Salience of consequences | 18 |
|  |  |  |  | Persuasion | 5.3 Information about social and environmental consequences | 17 |
|  |  |  |  |  | 5.6 Information about emotional consequences | 16 |
|  |  |  |  |  | 6.3 Information about other's approval | 11 |
|  |  |  |  |  | 8.1 Behavioral practice/rehearsal | 17 |
|  |  |  |  |  | 8.3 Habit formation | 16 |
|  |  |  |  |  | 9.2 Pros and cons | 16 |
|  |  |  |  |  | 11.2 Reduce negative emotions | 15 |
|  |  |  |  |  | 13.2 Framing/reframing | 19 |
