# Supplementary material for: Baby Buddy App for Breastfeeding and Behavior Change: Retrospective Study of the App Using the Behavior Change Wheel
Source: JMIR Mhealth Uhealth. 2021 Apr 15;9(4):e25668. doi: 10.2196/25668 (PMC8085747; doi:10.2196/25668)
Supplement: Multimedia Appendix 3 [file mhealth_v9i4e25668_app3.docx]

**Multimedia Appendix 3. Complete analysis of all breastfeeding items.**

|  | **Capability** | | **Opportunity** | | **Motivation** | |
| --- | --- | --- | --- | --- | --- | --- |
|  | *Physical* | *Psychological* | *Social* | *Physical* | *Reflective* | *Automatic* |
| **Video title** |  |  |  |  |  |  |
| Breastfeeding as a young mum | ✓ | ✓ | ✓ | ✓ | ✓ | ✓ |
| A practical choice | ✓ | ✓ | ✓ | ✓ | ✓ | ✓ |
| Feelings about breastfeeding | ✓ | ✓ | ✓ | ✓ | ✓ | ✓ |
| What’s so good about breastfeeding? |  | ✓ | ✓ | ✓ | ✓ | ✓ |
| What if I bottlefed before? | ✓ | ✓ | ✓ | ✓ | ✓ | ✓ |
| Asking for help to get started | ✓ | ✓ | ✓ | ✓ |  |  |
| What will my partner think? |  |  | ✓ |  | ✓ |  |
| Your first milk - colostrum | ✓ | ✓ |  |  | ✓ | ✓ |
| Your baby’s first feed | ✓ | ✓ | ✓ | ✓ |  | ✓ |
| Skin to skin | ✓ | ✓ | ✓ | ✓ |  | ✓ |
| Good positioning tips from a midwife | ✓ |  | ✓ |  |  | ✓ |
| Getting the position right | ✓ |  |  | ✓ |  | ✓ |
| Good positioning demonstration | ✓ | ✓ |  | ✓ | ✓ |  |
| Keeping your baby close | ✓ | ✓ | ✓ | ✓ | ✓ | ✓ |
| How dads can help - Lenny |  |  | ✓ | ✓ |  |  |
| Breastfeeding out and about |  | ✓ | ✓ |  | ✓ | ✓ |
| When and how often should I feed my baby? | ✓ | ✓ | ✓ | ✓ | ✓ | ✓ |
| How dads can help - Andy |  |  | ✓ | ✓ | ✓ |  |
| Where can I find support? | ✓ | ✓ | ✓ | ✓ | ✓ |  |
| Overcoming mastitis | ✓ | ✓ | ✓ | ✓ | ✓ | ✓ |
| Support from health professionals | ✓ | ✓ | ✓ | ✓ | ✓ | ✓ |
| Some common challenges | ✓ | ✓ |  | ✓ | ✓ |  |
| Good and bad attachment graphic | ✓ |  |  | ✓ |  |  |
| Breastfeeding to a year and beyond | ✓ | ✓ | ✓ | ✓ | ✓ | ✓ |
| Why breastfeed for at least six months? | ✓ | ✓ | ✓ | ✓ | ✓ | ✓ |
| Breastfeeding and weening | ✓ | ✓ | ✓ | ✓ | ✓ | ✓ |
| Why express? | ✓ | ✓ | ✓ | ✓ | ✓ | ✓ |
| How to hand express | ✓ | ✓ |  | ✓ | ✓ | ✓ |
| How to use a breast pump | ✓ |  |  | ✓ | ✓ |  |
| Expressing when you’re back at work | ✓ | ✓ |  | ✓ | ✓ |  |
| Storing and using expressed breast milk |  |  |  | ✓ | ✓ |  |
| Early challenges with expressing milk | ✓ | ✓ |  | ✓ | ✓ |  |
| Your breast milk | ✓ | ✓ | ✓ | ✓ | ✓ | ✓ |
| How skin-to-skin contact can help you express | ✓ | ✓ | ✓ | ✓ | ✓ | ✓ |
| Using a breast pump | ✓ |  | ✓ | ✓ | ✓ |  |
| Expressing with a breast pump and storing your milk | ✓ | ✓ | ✓ | ✓ | ✓ | ✓ |
| Colostrum – your baby’s first food | ✓ | ✓ |  | ✓ |  | ✓ |
| Signs your baby is ready to feed independently |  | ✓ | ✓ | ✓ | ✓ |  |
| Breastfeeding twins or triplets | ✓ |  |  | ✓ | ✓ |  |
| **Text responses to 'What does that mean?'** |  |  |  |  |  |  |
| Blocked ducts | ✓ |  |  | ✓ |  |  |
| Colostrum | ✓ |  |  |  |  |  |
| Expressing milk | ✓ |  |  | ✓ |  |  |
| Mastitis | ✓ |  |  | ✓ |  |  |
| Rooting reflex | ✓ |  |  |  |  |  |
| Reflex | ✓ |  |  |  |  |  |
| Skin-to-skin | ✓ | ✓ | ✓ | ✓ |  |  |
| Sterilising | ✓ |  |  | ✓ |  |  |
| **Text responses to questions - 'Ask me'** |  |  |  |  |  |  |
| What is expressing milk? | ✓ |  |  | ✓ |  |  |
| How do I express milk? | ✓ |  |  | ✓ |  |  |
| Can I carry on breastfeeding after returning to work, college or school? | ✓ |  |  | ✓ |  |  |
| Why do people say 'breast is best'? | ✓ | ✓ | ✓ |  | ✓ |  |
| What do I need to know about breastfeeding? |  |  |  | ✓ |  |  |
| How do I breastfeed when I'm out and about? | ✓ | ✓ | ✓ | ✓ | ✓ |  |
| Why is breastfeeding so good for babies and mums? | ✓ | ✓ | ✓ | ✓ | ✓ |  |
| I'm breastfeeding. Do I need to wake my newborn to feed? | ✓ |  |  | ✓ | ✓ |  |
| What is latching on? | ✓ |  |  | ✓ |  | ✓ |
| Why are my breasts growing and sore? | ✓ |  |  | ✓ |  |  |
| How can I mix breastfeeding and bottlefeeding? | ✓ | ✓ | ✓ | ✓ |  |  |
| How do I know if my baby is hungry? |  |  |  | ✓ |  | ✓ |
| How often should I feed my baby? | ✓ |  | ✓ | ✓ |  |  |
| What is colostrum? | ✓ |  |  |  |  |  |
| What is milk 'coming in'? | ✓ |  |  | ✓ |  |  |
| What is skin-to-skin? | ✓ | ✓ | ✓ | ✓ |  | ✓ |
| What are blocked ducts and mastitis? | ✓ |  |  | ✓ |  |  |
| What are the different kinds of breast pumps? | ✓ |  |  | ✓ |  |  |
| What is baby-led attachment? | ✓ |  |  | ✓ |  | ✓ |
| How do I know my baby is getting enough milk? | ✓ |  |  | ✓ |  |  |
