# Supplementary material for: Baby Buddy App for Breastfeeding and Behavior Change: Retrospective Study of the App Using the Behavior Change Wheel
Source: JMIR Mhealth Uhealth. 2021 Apr 15;9(4):e25668. doi: 10.2196/25668 (PMC8085747; doi:10.2196/25668)
Supplement: Multimedia Appendix 4 [file mhealth_v9i4e25668_app4.docx]

**Multimedia Appendix 4. APEASE (affordability, practicability, effectiveness, affordability, safety, and equity) criteria.**

| Criterion | Judgement of effectiveness compared to existing resource | Evidence and analysis |
| --- | --- | --- |
| Acceptability | Baby Buddy is an acceptable intervention and extension of the existing breastfeeding website and magazine resources to users, implementers and funders. | Extensive consultation with stakeholders; implementers, funders and users was undertaken.  **Implementers and funders**   - In 2007 a collaboration between Best Beginning, the Department of Health and UNICEF, resulted in the ‘Bump to Breastfeeding’ web resources and the express magazine. - Over 2 million copies of the DVD were distributed.   **Users – mums, dads, support people and midwives**   - 2012 end user research was conducted to assess potential users’ response to the concept of the app and to gauge their reactions to initial ideas for the content/functions, visual style and tone. - Focus groups and semi-structured interview were conducted to explore the socio-ecological constructs that drive societal, family and individual breastfeeding behaviours. Participants included young parents/pregnant couples and student midwives   The main findings were:  • that Baby Buddy was easier to use and more accessible than books  • Baby Buddy is a ‘one stop shop’ with everything related to pregnancy in one place  • The app is fun to use and engaging  • The regular and relevant, ‘bitesize’ pieces of information were appealing  • Baby Buddy is easy to access 24/7 when midwives/support may not be available  • Baby Buddy provided up-to-date information from reliable sources  •The app acts as a reminder for appointments  In 2014 research was conducted to test the app prototype, gauge response to design, look, feel and fuctionality. Focus groups and a paired depth interviews with parents and pregnant women (aged 19-24)  suggest that partipants like the app design, sense of fun and youthfulness yet feel that it maintains a professional, credible and informative approach.  Researchers concluded that participants felt that the app was intuitive/easy to use and had a range of functions that were highly appealing, practical and more fun then other apps. |
| Practicability | Baby Buddy can be implemented at scale effectively to reach the target audience. The geo-location functionality makes the intervention more practical and can be updated quickly adding to reach. | Data was examined to determine the extent of use by the target population and the reach of smartphones in city and regional areas of the UK. A case study evalution of the pilot app was undertaken and reviewed. National in-app data & in-app data from Guys and St Thomas’ and Blackpool was collected between 19 November 2014 to 19 May 2015 and reported [[24](#_ENREF_24)]. Baby Buddy was successful in reaching its target demographic. A total of 80% of users register as "mums" and a high number of registrations coming from younger users [[22](#_ENREF_22)].  Geo-location functionality also provides web address, location, phone numbers, email addresses, opening times for hospitals, GP surgeries, drop in centres and groups and play centres. These provide further support and information related to breastfeeding. Users can also recommend services that they use in their local area and customise. |
| Effectiveness | Academic evaluations support that embedding Baby Buddy into maternity and early years care is feasible, attractive and can be beneficial for maternal and infant health [[25](#_ENREF_25)]. | In 2014 two pieces of research looked at the effectiveness of Baby Buddy The aims of the research were:  • To understand where young mums and mums-to-be get their pregnancy/new baby information currently, and ascertain whether they have all their information/advice needs met.  • To explore experiences of using the Baby Buddy app amongst the target audience of younger mums and mums-to-be, specifically: appearance, navigation, functionality, content, unlocking content/rewards.  •To get feedback on the preferred directional style for films within the app.  •To assess effectiveness of the reactions to the Baby Buddy leaflet.  Focus groups were conducted, five key insights emerged from the research:  1) Although there is a lot of information and advice about pregnancy available, most young mums and mums-to-be felt that the Baby Buddy app was unique, new and exciting. Three core strengths of the app were identified:  • A definitive and trustworthy source of advice  • Personal and tailored to you  • Engaging and entertaining as well as informative  2) The avatar is central to the appeal of the app and differentiates it from other apps on the market. The degree to which young mums and mums-to-be engage with the avatar varies.  3) Of the other features, ‘Today’s information’ and ‘Videos’ are most popular, followed by ‘Ask me’, ‘What does that mean’.  4) ‘You can do it is the least attractive feature’. Although most young mums and mums-to-be acknowledge the importance of a healthier lifestyle, many are resistant to being told what to do.  5) Young mums and mums-to-be like the idea of being incentivised/rewarded for giving their feedback. Whilst accessories for the avatar work for those who engage fully with the avatar concept, only real world rewards are motivating to others.  The extent of effectiveness of the app as a behaviour change intervention for breastfeeding, was measured in an independent research report by the University of Central Lancashire (2017). The aim of the project was to explore the impact of the embedding and usage of the resources on breastfeeding rates, women’s decisions, attitudes and self-efficacy in relation to breastfeeding. Women’s confidence in parenting, and mother–infant relationships as well as trying to understand how the embedding process had worked including barriers and facilitators [[25](#_ENREF_25)]. Results showed that for some women the resources had increased their knowledge of infant feeding and reinforced their decision to breastfeed [[25](#_ENREF_25)].  An independent report by Crossland et al (2017) was commissioned [18]. Baby Buddy app was rated highly by women and professionals. In the women’s survey, 81/117 (69%) respondents rated the quality of the Baby Buddy app as ‘excellent’, ‘very good’ or ‘good’, and in the professionals’ survey, 128/146 (88%) of professionals rated the Baby Buddy app as either excellent, very good or good. |
| Affordability | Findings suggest that because the app is free and downloads are able to be saved to the device, most women would not see affordability as a barrier. | This complete resource is free to download and there is no option for in-app purchases. Downloading is dependent on particular smartphone or tablet technology, namely Android 4.0 or iOS 7.0 or above. It also requires 32 MB of storage [[22](#_ENREF_22)].  Best Beginnings as a charity and depends on grants, donations and industry support for it to be financially viable. Endorsement and key partnerships with the NHS, Universities and the royal family have led to funding opportunities and are key to the sustainability of Baby Buddy. Best Beginings is able to operate without the commericalisation that is seen in other popular apps.  Best Beginnings has circumvented social and contextual barriers, as smartphone ownership and internet access are variable and unequal, by ensuring that Baby Buddy is free and included films are available to download in wifi zones. The films can be saved to watch offline later [[22](#_ENREF_22)]. |
| Safety | Baby Buddy if used as a complementary source of support and information in conjuction with face-to-face consultation, is safe for the target audience. We concluded that the apps is evidence-based and follows best practice with respect to health literacy and cultural safety. | Baby Buddy is unlikely to have unwanted effects as the content is evidence based and guided by best practice at a health literacy level of age 11.  This is supported by Powells’ et al (2016) who found that women felt that the app provided a source of credible information and supported where. the women encountered competing or conflicting advice. Midwives felt that Baby Buddy providing a ‘backup’ when they were not around and complementary to their knowledge and expertise, specialist information, advice and support for expectant and new mothers [[22](#_ENREF_22)]. |
| Equity | Findings from Cooper, (2015), Powell et al (2016), and Crossland et al (2017) showed that Baby Buddy is broadely accessibility with the content written in such a way that it can be understood by anyone with a reading age of 11 or above and also has a ‘read aloud’ option. Powell et al (2016) found that Baby Buddy also applied to ethnicity as a sociodemographic variable as text can also be translated. | This intervention primarily is aimed at aims to be available to all socio-economic groups and aims to address health inequalities. The design of Baby Buddy is based on a theory of ‘proportionate universality’ for health interventions [[26](#_ENREF_26)]. Although Baby Buddy is intended to benefit a wide range of app users, its design has been styled to be particularly attractive to expectant mothers who are under 25 years of age. Data shows that Baby Buddy is reaching women of all ages and is more successful at attracting younger mums [[22](#_ENREF_22)]. |
